# RETRACTION: Histone Lactylation‐Driven Upregulation of VRK1 Expression Promotes Stemness and Proliferation of Glioma Stem Cells

**DOI:** 10.1002/advs.74235

**Published:** 2026-02-03

## Abstract

**RETRACTION**: J. Li, C. Tang, X. Zhang, R. Xing, and Q. Guo, “Histone Lactylation‐Driven Upregulation of VRK1 Expression Promotes Stemness and Proliferation of Glioma Stem Cells,” *Advanced Science* 12, no. 44 (2025): e03897, https://doi.org/10.1002/advs.202503897.

The above article, published online on 24 September 2025, in Wiley Online Library (http://onlinelibrary.wiley.com/), has been retracted by agreement between the authors; the journal Editor‐in‐Chief, Kirsten Severing; and Wiley‐VCH GmbH. Following publication, the authors notified the journal that they had discovered an unintentional statistical error introduced during the gene‐screening process using multiple databases for lactate‐regulated glioma stem cell stemness. Specifically, when re‐analyzing the GSE145699 dataset to nominate candidate genes, the authors observed that VRK1 does not consistently reach statistical significance when different pairing strategies are applied in paired t‐tests. This instability undermines the reliability of the gene selection step (noted in Results 2.2) and cannot be rectified by a correction. These results were critical to the main interpretation of the data presented in the article, and as such, the article must be retracted.

**RETRACTION**: J. Li, C. Tang, X. Zhang, R. Xing, and Q. Guo, “Histone Lactylation‐Driven Upregulation of VRK1 Expression Promotes Stemness and Proliferation of Glioma Stem Cells,” *Advanced Science* 12, no. 44 (2025): e03897, https://doi.org/10.1002/advs.202503897.

The above article, published online on 24 September 2025, in Wiley Online Library (http://onlinelibrary.wiley.com/), has been retracted by agreement between the authors; the journal Editor‐in‐Chief, Kirsten Severing; and Wiley‐VCH GmbH. Following publication, the authors notified the journal that they had discovered an unintentional statistical error introduced during the gene‐screening process using multiple databases for lactate‐regulated glioma stem cell stemness. Specifically, when re‐analyzing the GSE145699 dataset to nominate candidate genes, the authors observed that VRK1 does not consistently reach statistical significance when different pairing strategies are applied in paired t‐tests. This instability undermines the reliability of the gene selection step (noted in Results 2.2) and cannot be rectified by a correction. These results were critical to the main interpretation of the data presented in the article, and as such, the article must be retracted.

